# A Mendelian randomization analysis reveals the role of the skin microbiota in systemic lupus erythematosus

**DOI:** 10.1007/s10067-025-07556-z

**Published:** 2025-07-05

**Authors:** Yangqi Yin, Jingting Zhao, Zhongbin Tao, Jie Wang

**Affiliations:** 1 Department of Pediatrics, General Hospital of Fushun Mining Bureau of Liaoning Health Industry Group, 113008 Fushun, China; 2 Department of Dermatology, The First Hospital of China Medical University, Shenyang, 110001 China; 3 Department of Pediatrics, The First Hospital of Lanzhou University, Lanzhou, 730000 China; 4 Department of Pediatrics, The Second People’s Hospital of Gansu Province, Lanzhou, 730000 China

**Keywords:** Genetic approaches, Mendelian randomization, Skin microbiota, Systemic lupus erythematosus

## Abstract

**Background:**

Systemic lupus erythematosus (SLE) is influenced by a complex array of factors, encompassing genetic, hormonal, and microbial components. This study seeks to investigate the causal relationships between specific skin microbiotas and SLE using a Mendelian randomization (MR) analysis and a Bayesian weighted Mendelian randomization (BWMR) analysis.

**Methods:**

We utilized genome-wide association study (GWAS) data to investigate the role of skin microbiota in SLE. Single nucleotide polymorphisms (SNPs) were employed as instrumental variables (IVs) in 9 Mendelian randomization methods, including the inverse variance weighted (IVW) method, MR-Egger method, weighted median method, weighted mode, simple mode method, contamination mixture (ConMix) method, robust adjusted profile score (RAPS) method, constrained maximum likelihood and model averaging (CML-MA), and debiased inverse-variance weighted (dIVW) method. Additionally, sensitivity analyses such as leave-one-out analysis, Cochran’s *Q* test, and Egger intercept test were conducted to ensure the robustness of the Mendelian randomization results. Finally, we applied Bayesian weighted Mendelian randomization (BWMR) approach to validate our MR findings.

**Results:**

In the MR analysis of the skin microbiota (KORA FF4) and SLE, the IVW method revealed that ASV042 (*Acinetobacter* (unc.)) _Antecubitalfossa_Moist (OR = 0.964, 95% CI = 0.941–0.988, *p* = 0.003) showed protective effects against SLE, while three taxa exhibited positive associations with SLE risk: ASV005 (*Propionibacterium granulosum*) _Retroauricularfold_Sebaceous (OR = 1.051, 95% CI = 1.013–1.091, *p* = 0.008), the phylum *Proteobacteria* _Retroauricularfold_Sebaceous (OR = 1.048, 95% CI = 1.004–1.094, *p* = 0.033), and the class *Betaproteobacteria* _Retroauricularfold_Sebaceous (OR = 1.049, 95% CI = 1.00 l–1.098, *p* = 0.044). In the MR analysis of the skin microbiota (PopGen) and SLE, five taxa demonstrated protective effects: ASV004 (*Corynebacterium* (unc.)) _Forehead_Sebaceous (OR = 0.960, 95% CI = 0.927–0.994, *p* = 0.023), ASV005 (*Propionibacterium granulosum*) _Antecubitalfossa_Moist (OR = 0.964, 95% CI = 0.930–0.999, *p* = 0.042), ASV007 (*Anaerococcus* (unc.)) _Forehead_Sebaceous (OR = 0.969, 95% CI = 0.942–0.996, *p* = 0.027), the genus *Kocuria* _Volarforearm_Dry (OR = 0.964, 95% CI = 0.935–0.995, *p* = 0.023), and the class *Betaproteobacteria* _Antecubitalfossa_Moist (OR = 0.952, 95% CI = 0.918–0.988, *p* = 0.010), while ASV039 (*Acinetobacter* (unc.)) _Antecubitalfossa_Moist (OR = 1.024, 95% CI = 1.000–1.048, *p* = 0.049) was found to potentially increase risk in SLE. The findings were further supported by BWMR analysis, adding credibility to this research. Sensitivity analyses confirmed robustness of these findings.

**Conclusion:**

Through genetic approaches, our study has illustrated specific skin microbiotas that may influence SLE, potentially facilitating earlier detection and enhancing the efficacy of treatment alternatives for patients with SLE.

**Supplementary Information:**

The online version contains supplementary material available at 10.1007/s10067-025-07556-z

**Table:** 

**Key points** • *This is the first study to uncover the potential causal relationship between skin microbiota and SLE using MR analysis.* • *The study has identified a potential causal relationship between 10 skin microbiotas and SLE*. • *A series of sensitivity analyses and BWMR were conducted to validate the robustness of our findings.*	

Systemic lupus erythematosus (SLE) is a chronic inflammatory autoimmune disease which may impart multiple organ systems including the skin, joints, hematological system, nervous system, and internal organs. In a meta-analysis of 112 studies, the incidence of SLE was estimated to be 5.14 (1.4–15.13) per 100,000 person-years, with approximately 400,000 new cases of SLE diagnosed annually; it is estimated to have a prevalence of 43.7 (15.87 to 108.92) per 100,000 individuals and impacts approximately 3.41 million people globally [1]. A national retrospective study conducted in Hungary revealed that the standardized mortality rates for individuals with SLE and those receiving treatment for SLE were 1.63 and 2.09, respectively, both significantly exceeding those of the general population [2]. Currently, the management of SLE predominantly relies on prolonged administration of corticosteroids, conventional disease-modifying antirheumatic drugs (DMARDs), and biologic DMARDs. Many of these medications are associated with significant adverse effects, such as the potential for corticosteroids to induce osteoporosis, cataracts, and avascular necrosis with prolonged use [3], cyclophosphamide to cause hemorrhagic cystitis and premature ovarian failure [4], and Belimumab to increase the risk of serious psychiatric adverse events [5]. Hence, there is a pressing necessity to delve deeper into the etiology of systemic lupus erythematosus in order to discover novel strategies for its prevention and management.

Recent research has indicated that the development of SLE is attributed to a complex interplay of genetic, hormonal, and environmental factors in individuals who are predisposed to the condition. Among environmental factors, the microbiota has been associated with the pathogenesis of SLE. Previous Mendelian randomization (MR) studies have revealed causal effects of gut microbiome on SLE [6]. The skin serves as the primary organ implicated in SLE, with approximately 70–85% of SLE patients presenting with cutaneous manifestations [7]. The skin microbiota has also been implicated in the pathogenesis of various inflammatory skin conditions, including psoriasis, vitiligo, and atopic dermatitis [8]. Previous research has demonstrated that application of *Staphylococcus aureus* on epithelial cell–specific IκBζ deficient (*Nfkbiz*^ΔK5^) mice led to the development of SLE-like manifestations in these mice [9]. Therefore, the skin microbiota is believed to play a pivotal role in the pathogenesis of SLE. It is necessary to use MR methods to reveal the causal effect of skin microbiota on SLE. We aim to provide new insights into SLE pathophysiology and reveal potential targets for future targeted drug development.

## Materials and methods

### Ethical considerations

Our study utilized data from publicly accessible databases, and no additional ethical approval was deemed necessary.

### Study design

**Fig. 1:**
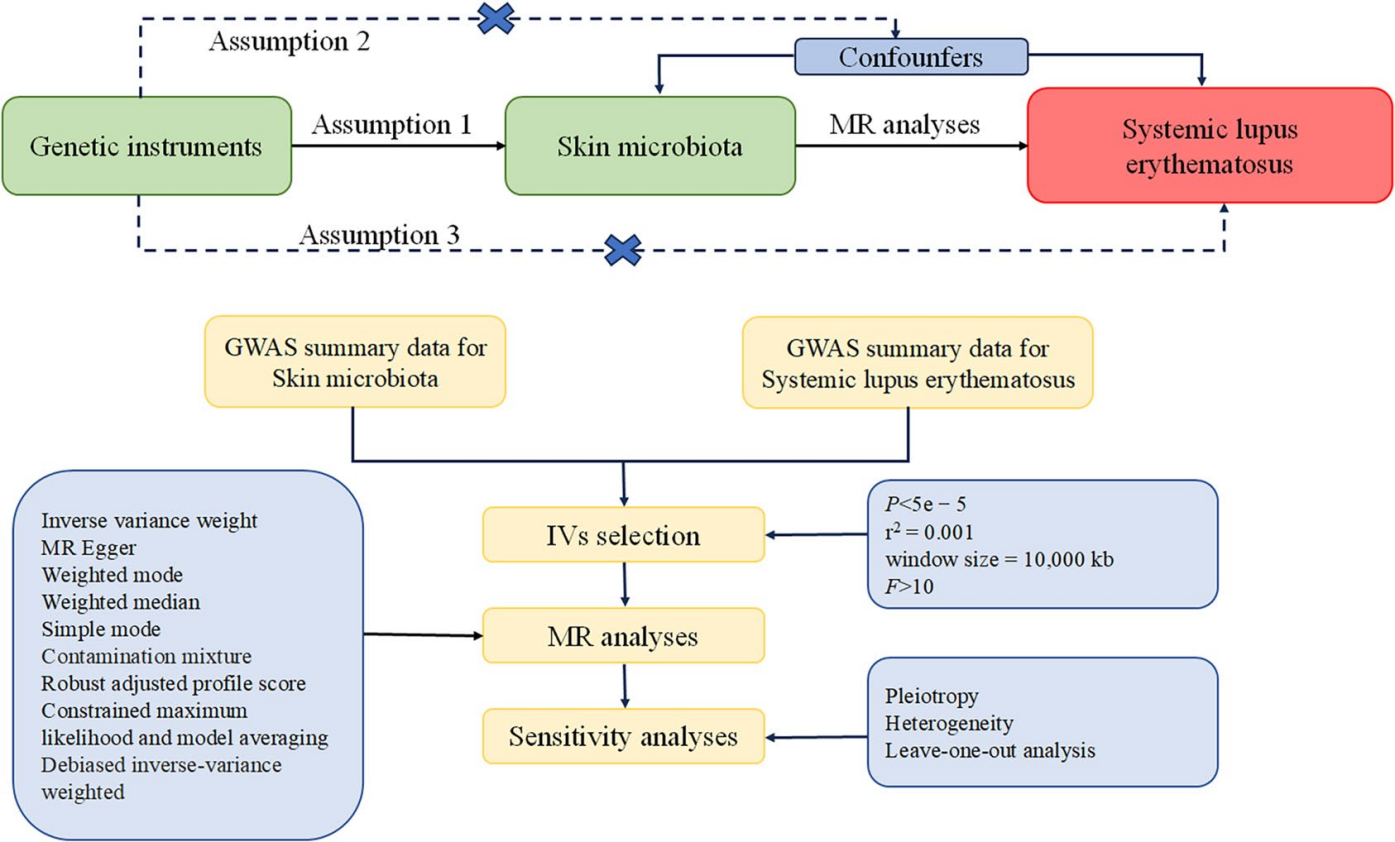
A comprehensive overview of the study design, encompassing the underlying assumptions of the Mendelian randomization (MR) study and a schematic representation of the MR analysis

Our MR analysis is structured on three underlying assumptions. Firstly, the relevance assumption involved utilizing single nucleotide polymorphisms (SNPs) closely associated with exposures as instrumental variables (IVs). Secondly, the independence assumption stated that these IVs showed no correlation with relevant confounding factors. Lastly, the exclusivity assumption proposed that these IVs influenced outcomes through their effects on exposure exclusively [10–12]. A detailed outline of the research design is depicted in Fig. 1, which encompasses the fundamental assumptions of the MR study and an illustration of the MR analysis.

### Data sources

The GWAS data for skin microbiota were collected from two German cohorts, KORA FF4 (*n* = 324) [13] and PopGen (*n* = 273) [14], as outlined in the summary by Moitinho-Silva et al. [8]. The KORA FF4 cohort exhibited a slight female predominance (47% male vs 53% female), whereas the PopGen cohort demonstrated a male-skewed distribution (58% male vs 42% female). Age distributions differed significantly between cohorts: KORA FF4 participants were exclusively middle-aged (39–48 years), whereas PopGen encompassed predominantly older adults (50–80 years). A total of 1656 skin samples were analyzed, collected from different microenvironments including the dry areas of the dorsal and volar forearm, the moist area of the antecubital fossa, and the sebaceous regions of the retro-auricular fold and forehead. The characteristics of skin microbiota were categorized based on various levels such as phylum, class, order, family, genus, and amplicon variant sequences (AVS), in addition, uncultured (unc.), indicating that the specific microorganism has not been successfully cultured in a laboratory environment.

The GWAS summary data for SLE was acquired from the FinnGen consortium’s website (https://www.finngen.fi/fi) on July 10th, 2024. The GWAS ID is finngen_R11_SLE_FG. Within this dataset were 1198 confirmed cases of SLE and 338,286 individuals with Finnish ancestry who served as controls.

### The selection of instrumental variables

To identify appropriate SNPs as IVs for subsequent investigation, we implemented the following set of screening criteria. Firstly, a significance threshold of *p* < 5e-5 was utilized to exclude SNPs lacking significant associations with exposure. Subsequently, the linkage disequilibrium (LD) coefficient *r*^2^ should be less than 0.001, and the clumping window size should exceed 10,000 kb to ensure independence of the SNPs. Finally, *F*-statistics for these IVs were computed to mitigate bias arising from weak instrumental variables. A value of *F*-statistics exceeding 10 suggests the strong presence of IVs [11].

### MR analysis

In our analysis of MR, we employed 9 different methods to evaluate the causal impact of the skin microbiota on SLE. Our analysis primarily utilized the random-effects inverse-variance weighted (IVW) approach, which aggregates Wald ratio estimates across multiple genetic variants through meta-analysis. This method delivers maximum precision under the assumption of valid IVs [15–17]. For supplementary analysis, we implemented the weighted median approach, which maintains consistency when over half of the weighted information comes from valid IVs [18]. The analytical framework also incorporated MR-Egger regression, employing an IVW linear regression model that regresses outcome associations against exposure associations [11]. Although useful for identifying directional pleiotropy, this technique shows particular sensitivity to outlying variants and often yields less precise estimates [19]. While the simple mode approach has lower statistical efficiency relative to IVW, its model-based framework confers particular advantages in addressing pleiotropic effects [20]. The weighted mode estimator remains effective when a majority of IVs are valid, demonstrating robustness even in the presence of some invalid IVs that violate standard MR assumptions [21]. The contamination mixture method (ConMix) represents an innovative bidirectional approach for MR analysis. This method operates through two key mechanisms: clustering genetic variants with comparable causal estimates to identify potential distinct biological pathways mediating the exposure-outcome relationship and providing robust causal inference even when invalid instruments are present. Simulation studies demonstrate that ConMix outperforms alternative robust MR methods, achieving superior precision as evidenced by consistently lower mean squared error across diverse realistic scenarios [22]. To address both correlated and uncorrelated pleiotropic effects while avoiding the Instrument Strength Independent of Direct Effect (InSIDE) assumption, we implemented constrained maximum likelihood with model averaging (CML-MA) [23]. Our analytical framework also incorporated robust adjusted profile score (RAPS) regression, which effectively handles both systematic and idiosyncratic pleiotropy while maintaining reliable inference under conditions of widespread weak instrument bias [24]. Additionally, the debiased IVW (dIVW) estimator was employed to minimize bias from heterogeneous instrument effects, particularly valuable when analyzing variants with varying effect sizes [25].

The main analytical technique employed was the IVW method, and variables with a significance level below *p* < 0.05 were chosen for further analysis [26]. To further bolster the strength of causal inference, we employed Bayesian weighted Mendelian randomization (BWMR) in our study. The BWMR model considers the uncertainty resulting from polygenicity and deals with breaches of IV assumptions caused by pleiotropy using Bayesian-weighted outlier detection [27]. In order to ensure the robustness and reliability of our findings, we conducted a series of sensitivity analyses to evaluate pleiotropy and heterogeneity. We utilized MR-PRESSO (MR-Pleiotropy RESidual Sum and Outlier) and MR-Egger intercept tests to monitor potential horizontal pleiotropy effects, with a significance level set at *p* < 0.05. Heterogeneity among the selected SNPs was assessed using Cochran’s *Q* statistic, and a systematic “leave-one-out” analysis was performed to identify any potentially heterogeneous SNPs by excluding each instrumental SNP one at a time. Additionally, we also employed the Steiger test to detect and correct for any bias resulting from reverse causality.

All statistical analyses were conducted using the TwoSampleMR and MR-PRESSO packages in R software (version 4.3.0).

## Results

### MR analysis

**Fig. 2:**
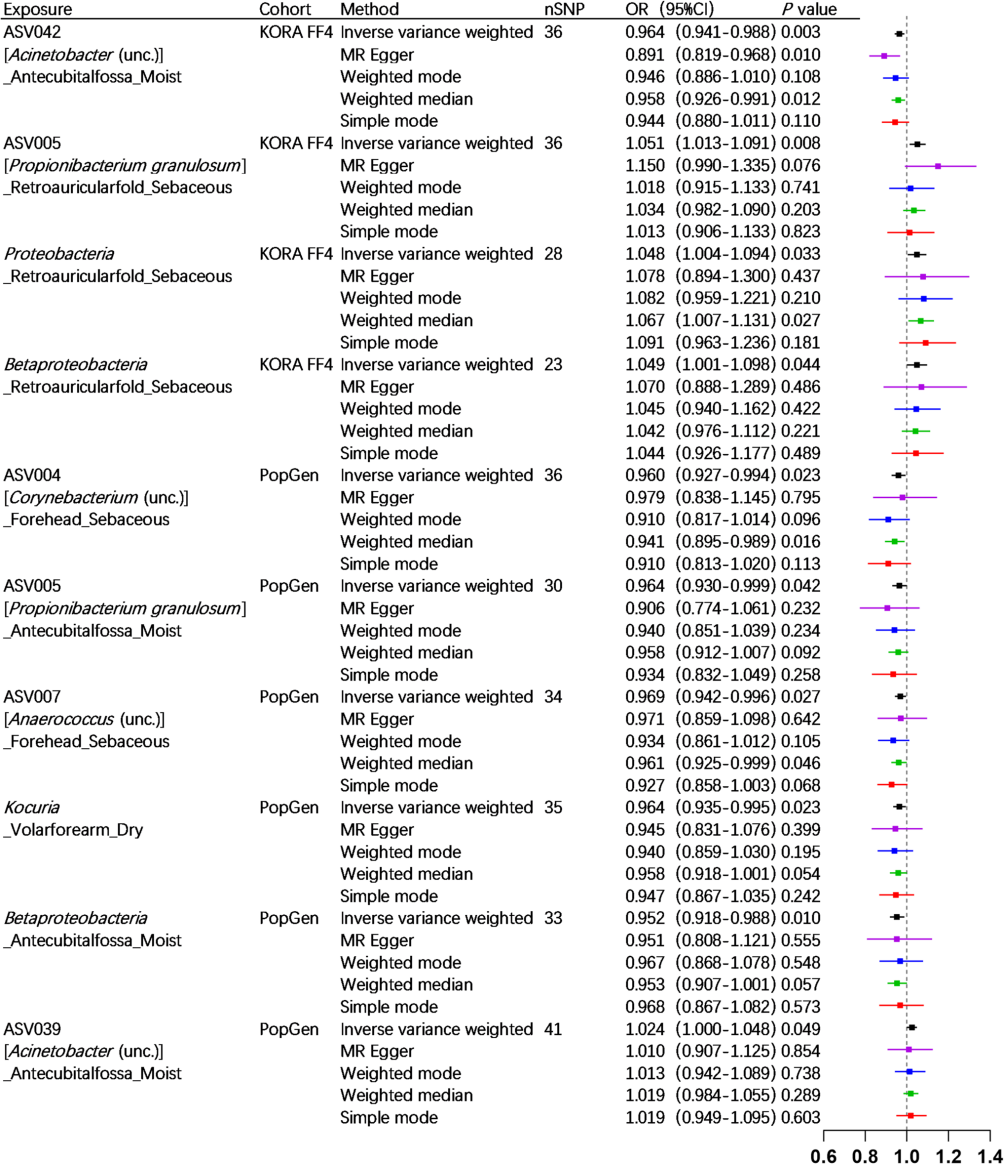
Forest plots of significant mendelian randomization (MR) analysis results. CI, confidence interval; OR, odds ratio; nSNP, the number of SNP; ASV, amplicon variant sequence; unc., uncultured

**Fig. 3:**
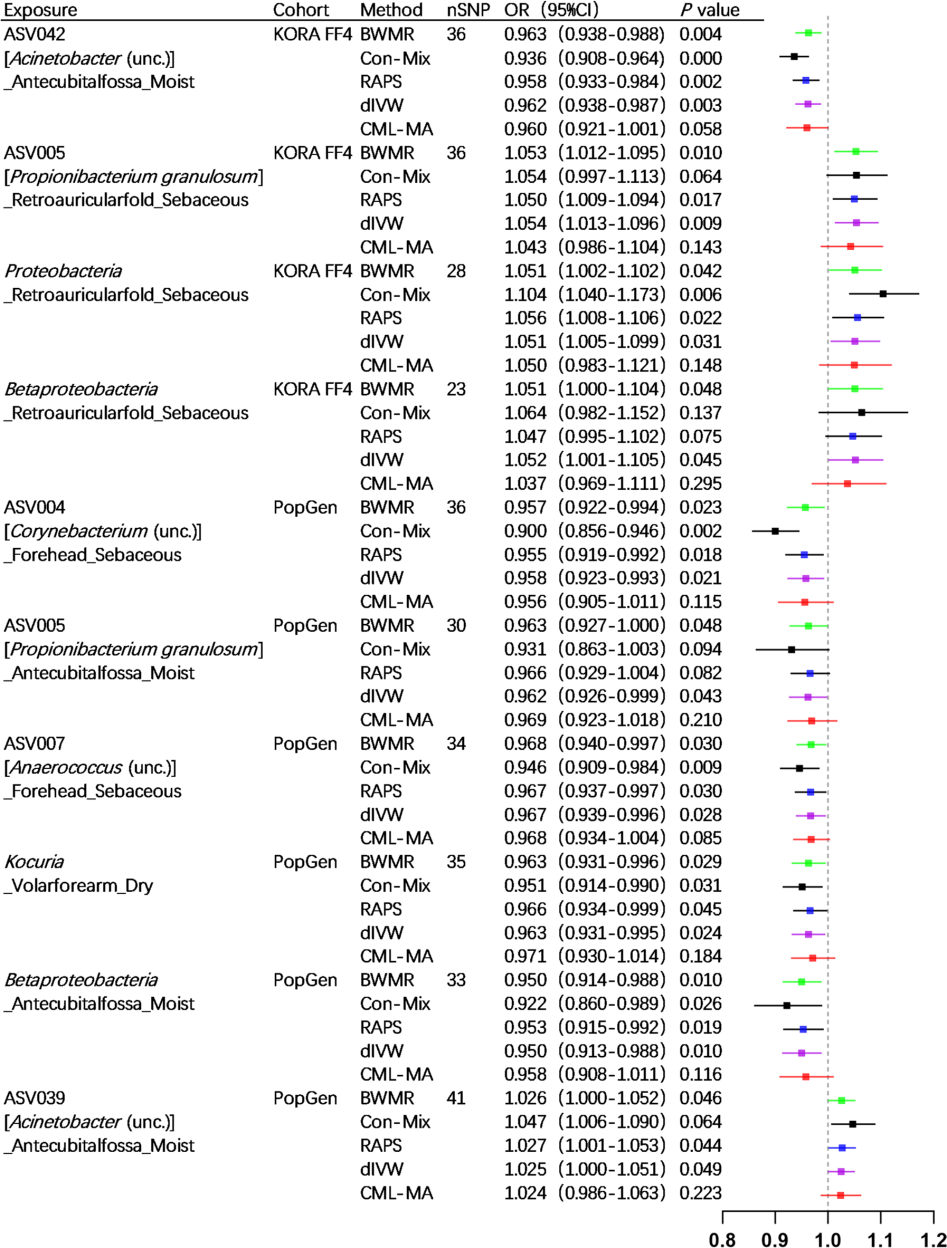
Forest plots of Bayesian weighted Mendelian randomization (BWMR) and significant mendelian randomization (MR) analysis results. CI, confidence interval; OR, odds ratio; nSNP, the number of SNP; ASV, amplicon variant sequence; unc., uncultured; BWMR, Bayesian weighted Mendelian randomization; Con-Mix, the contamination mixture; RAPS, the robust adjusted profile score; CML-MA, constrained maximum likelihood and model averaging; dIVW, debiased inverse-variance weighted

We employed 2682 SNPs as IVs for MR analysis in the KORA FF4 cohort, and 7534 SNPs for MR analysis in the PopGen cohort (detailed information provided in Supplementary Table S1 and Supplementary Table S2). The IVW analysis, serving as the primary method of analysis, has identified ten skin microbiotas that may potentially have a causal relationship with SLE. Specifically, in the KORA FF4 cohort, ASV042 (*Acinetobacter* (unc.)) _Antecubitalfossa_Moist (OR = 0.964, 95% CI = 0.941–0.988, *p* = 0.003) demonstrated a protective effect. Conversely, three exhibited positive associations with SLE risk: ASV005 (*Propionibacterium granulosum*) _Retroauricularfold_Sebaceous (OR = 1.051, 95% CI = 1.013–1.091, *p* = 0.008), the phylum *Proteobacteria* _Retroauricularfold_Sebaceous (OR = 1.048, 95% CI = 1.004–1.094, *p* = 0.033), and the class *Betaproteobacteria* _Retroauricularfold_Sebaceous (OR = 1.049, 95% CI = 1.00 l–1.098, *p* = 0.044). In the PopGen cohort, five microbial taxa exhibited protective associations: ASV004 (*Corynebacterium* (unc.)) _Forehead_Sebaceous (OR = 0.960, 95% CI = 0.927–0.994, *p* = 0.023), ASV005 (*Propionibacterium granulosum*) _Antecubitalfossa_Moist (OR = 0.964, 95% CI = 0.930–0.999, *p* = 0.042), ASV007 (*Anaerococcus* (unc.)) _Forehead_Sebaceous (OR = 0.969, 95% CI = 0.942–0.996, *p* = 0.027), the genus *Kocuria* _Volarforearm_Dry (OR = 0.964, 95% CI = 0.935–0.995, *p* = 0.023), and the class *Betaproteobacteria* _Antecubitalfossa_Moist (OR = 0.952, 95% CI = 0.918–0.988, *p* = 0.010). Conversely, ASV039 (*Acinetobacter* (unc.)) _Antecubitalfossa_Moist (OR = 1.024, 95% CI = 1.000–1.048, *p* = 0.049) was associated with increased SLE risk (details provided in Figs. 2 and 3). The findings were supported by the BWMR analysis (details provided in Fig. 3). The scatter plots illustrating the causal relationship between ten skin microbiotas and SLE are presented in Supplementary Figure S1 and Supplementary Figure S2.

### Sensitivity analysis

In order to evaluate potential bias in the MR analysis, a sequence of sensitivity analyses was performed, and the results of the heterogeneity test and pleiotropy test are presented in Supplementary Table S3, Supplementary Table S4, and Supplementary Table S5. The MR-PRESSO and MR-Egger intercept tests indicated no horizontal pleiotropy. The “leave-one-out” analysis did not identify any outliers, suggesting that the findings were robust and not influenced by individual gene variants (details provided in Supplementary Fig. S3 and Supplementary Fig. S4). Furthermore, Cochran’s *Q* statistic did not show significant evidence of heterogeneity (*p* > 0.05). The MR Steiger directionality test also yielded normal results.

## Discussion

This research aimed to investigate the potential causal link between the skin microbiota and SLE using MR analysis. Furthermore, we performed sensitivity analysis and leave-one-out analysis to mitigate the impact of confounding variables. Our MR analysis revealed significant causal relationships between ten specific skin microbiotas and SLE.

The study identified ASV042 (*Acinetobacter* (unc.)) may be a protective factor against SLE in the KORA FF4 cohort. The *Acinetobacter* species are ubiquitous in the environment and are characterized as aerobic, glucose-nonfermentative gram-negative bacteria. They are commonly regarded as benign commensal organisms residing on the skin. Recent studies have demonstrated that *Acinetobacter* of the skin is capable of inducing interleukin (IL)10 production by monocytes and T cells [28]. It has been observed that IL-10 can exhibit both anti-inflammatory and pro-inflammatory effects in the pathogenesis of SLE in mice. Furthermore, blocking anti-IL-10 antibodies prior to the onset of SLE has been shown to effectively delay the production of autoantibodies [29]. The potential protective effect of ASV042 (*Acinetobacter* (unc.)) in SLE may be attributed to its mechanism of action involving the modulation of IL-10. However, ASV039 (*Acinetobacter* (unc.)) was identified as a risk factor for SLE in the PopGen cohort. This suggests that various *Acinetobacter* species may have varying contributions to the pathogenesis of SLE, although further research is necessary to confirm these findings in light of the study’s limitations.

The study has identified ASV005 (*Propionibacterium granulosum*) and the class *Betaproteobacteria* as potential risk factors for SLE in the KORA FF4 cohort. *Propionibacterium granulosum* shares similarities with *Propionibacterium acnes* as both are commensal bacteria of the pilosebaceous glands. The potential pathogenic role of *Propionibacterium granulosum* in acne vulgaris has been hypothesized based on its identification from acne lesions, but a higher prevalence of *Propionibacterium granulosum* was observed in unaffected hair follicles of individuals with acne [30, 31]. Recent studies suggest that *Propionibacterium granulosum* has the potential to outcompete *Propionibacterium acnes* by producing an endogenous extracellular nuclease, BmdE, which is capable of breaking down the biofilm of *Propionibacterium acnes *[32]. A previous MR analysis indicated that the class *Betaproteobacteria* was associated with an increased risk of alopecia areata [33]. Certain skin infectious diseases are caused by *Betaproteobacteria*. For example, the *Betaproteobacteria* class includes *Burkholderia*, which is accountable for infectious skin disorders like melioidosis [34]; upon infecting the skin, it induces inflammasome activation and keratinocyte extrusion [35]. It is noteworthy that similar skin microbiota may exert contrasting effects on the pathogenesis of SLE in different populations. In the PopGen cohort, the study identified the class *Betaproteobacteria* and ASV005 (*Propionibacterium granulosum*) as protective factors for SLE, but they are risk factors in the KORA FF4 cohort. We speculate that this is related to the different age distribution and sex ratio of the two cohorts. In the KORA FF4 cohort, the age distribution of the population was predominantly centered between 39 and 48 years old, with a relatively high proportion of female participants. However, in the PopGen cohort, there was a more widely dispersed age distribution but primarily concentrated between 60 and 80 years old, with a relatively high proportion of female participants. This may explain the differences in SLE prevalence between cohorts with different ages and male to female ratios. SLE is more common in young women between the ages of 30 and 40. However, the incidence of SLE is significantly decreased in individuals over 50 years old, and the female predominance is also notably diminished. Previously, the unique pathogenetic feature of SLE was believed to be associated with estrogen levels [36, 37]; however, it now appears that the alteration in the role of certain skin microbiota in the pathogenesis of SLE is also a contributing factor.

Our study has identified the phylum *Proteobacteria* as a potential risk factor for SLE in the KORA FF4 cohort. The phylum *Proteobacteria*, one of the predominant phyla of skin microbiota, predominantly comprises *Acinetobacter* and *Methylobacterium*. The role of the phylum *Proteobacteria* in the skin in contributing to the development of SLE is still not fully understood. While it has been proposed that the presence of *Proteobacteria* in gut could potentially play a role in the pathogenesis of lupus nephritis by promoting IL-6 production [38].

The study identified ASV004 (*Corynebacterium* (unc.)), ASV007 (*Anaerococcus* (unc.)), and the genus *Kocuria* maybe protective factors against SLE in the PopGen cohort. *Corynebacterium* stands as one of the common bacterial genera residing on the human skin, particularly thriving in areas with high moisture levels [39]. Previous studies have shown that *Corynebacterium* enhances the proliferation and activation of specific γδT cell populations in the dermis of mice [39]. By coincidence, the number of γδT cells in peripheral blood of SLE patients is lower than that of healthy people [40], while the number of γδT cells is increased after treatment [41]. The protective effect of ASV004 (*Corynebacterium* (unc.)) on SLE may be attributed to its interaction with γδT cells; however, additional experiments are necessary to validate this hypothesis. The *Anaerococcus* refers to a common commensal bacterium of human skin. Certain *Anaerococcus*, such as *Anaerococcus* sp. A20, have been associated with human osmidrosis [42]. This study indicates that ASV007 (*Anaerococcus* (unc.)) may serve as a potential protective factor against SLE, and further investigation is warranted to elucidate the specific protective mechanism. The genus *Kocuria* consists of gram-positive coccoid bacteria that are classified within the family *Micrococcaceae *[43]. It is commonly found colonizing the skin in humans. A previous MR analysis suggested that the genus *Kocuria* was linked to a reduced risk of atopic dermatitis [44]. Recent research has shown that the *Kocuria* culture supernatant significantly hinders the growth and formation of biofilms by *Staphylococcus aureus *[44]; this has been proposed as a potential explanation for its function as a protective element in atopic dermatitis. Further research is needed to elucidate *Kocuria* how can prevent the development of SLE.

## Conclusion

This study emphasizes the significant role of skin microbiotas in SLE by using of MR analysis to identify specific microbial taxa may impact the pathogenesis of SLE. Protective and risk factors of the microbiota have been identified, offering potential assistance for early diagnosis and treatment targets for SLE. Similar skin microbiota may exert varying effects on the pathogenesis of SLE in different populations, potentially contributing to the significant differences in SLE incidence across age groups. Further research is necessary to elucidate the underlying mechanisms of these findings.

## Supplementary Information

**Supplementary Fig. S1** Scatter plot analysis of the association between skin microbiota (KORA FF4) and systemic lupus erythematosus. **Supplementary Fig. S2** Scatter plot analysis of the association between skin microbiota (PopGen) and systemic lupus erythematosus. **Supplementary Fig. S3** Forest plots of SNPs associated with skin microbiota (KORA FF4) and systemic lupus erythematosus. **Supplementary Fig. S4** Forest plots of SNPs associated with skin microbiota (PopGen) and systemic lupus erythematosus. **Supplementary Table S1.** Characteristics of SNPs serving as IVs in KORA FF4 cohorts. **Supplementary Table S2.** Characteristics of SNPs serving as IVs in PopGen cohorts. **Supplementary Table S3.** Cochrane’s Q test of identified microbial taxa with systemic lupus erythematosus. **Supplementary Table S4.** MR-Egger intercept test of identified microbial taxa with systemic lupus erythematosus. **Supplementary Table S5.** MR-PRESSO test of identified microbial taxa with systemic lupus erythematosus.

Below is the link to the electronic supplementary material.

ESM 1 (XLSX 1.55 MB)

ESM 2 (PDF 1.40 MB)

## Data Availability

The GWAS data utilized in this study are accessible to the public and can be acquired from GWAS Catalog (https://www.ebi.ac.uk/gwas/); FinnGen consortium’s website (https://www.finngen.fi/fi); the summary data examined in this study can be obtained from articles and supplementary materials.

## Abbreviations

SLE: Systemic lupus erythematosus
MR: Mendelian randomization
BWMR: Bayesian weighted mendelian randomization
GWAS: Genome-wide association studies
IVs: Instrumental variables
SNPs: Single nucleotide polymorphisms
IVW: Inverse variance weighted
Con-Mix: The contamination mixture
RAPS: The robust adjusted profile score
CML-MA: Constrained maximum likelihood and model averaging
dIVW: debiased inverse-variance weighted
MR-PRESSO: MR-Pleiotropy RESidual Sum and Outlier
OR: Odds ratio
CI: Confidence interval
DMARDs: Disease-modifying antirheumatic drugs
ASV: Amplicon variant sequence
LD: Linkage disequilibrium
IL: Interleukin
