# Supplementary material for: A Mendelian randomization analysis reveals the role of the skin microbiota in systemic lupus erythematosus
Source: Clin Rheumatol. 2025 Jul 5;44(8):3191–9. doi: 10.1007/s10067-025-07556-z (PMC12289761; doi:10.1007/s10067-025-07556-z)

Supplementary Fig. S1 Scatter plot analysis of the association between skin microbiota (KORA FF4) and systemic lupus erythematosus. (a) ASV042 [Acinetobacter (unc.)];(b) ASV005 [*Propionibacterium granulosum*];(c) the phylum *Proteobacteria*;(d) the class *Betaproteobacteria*.

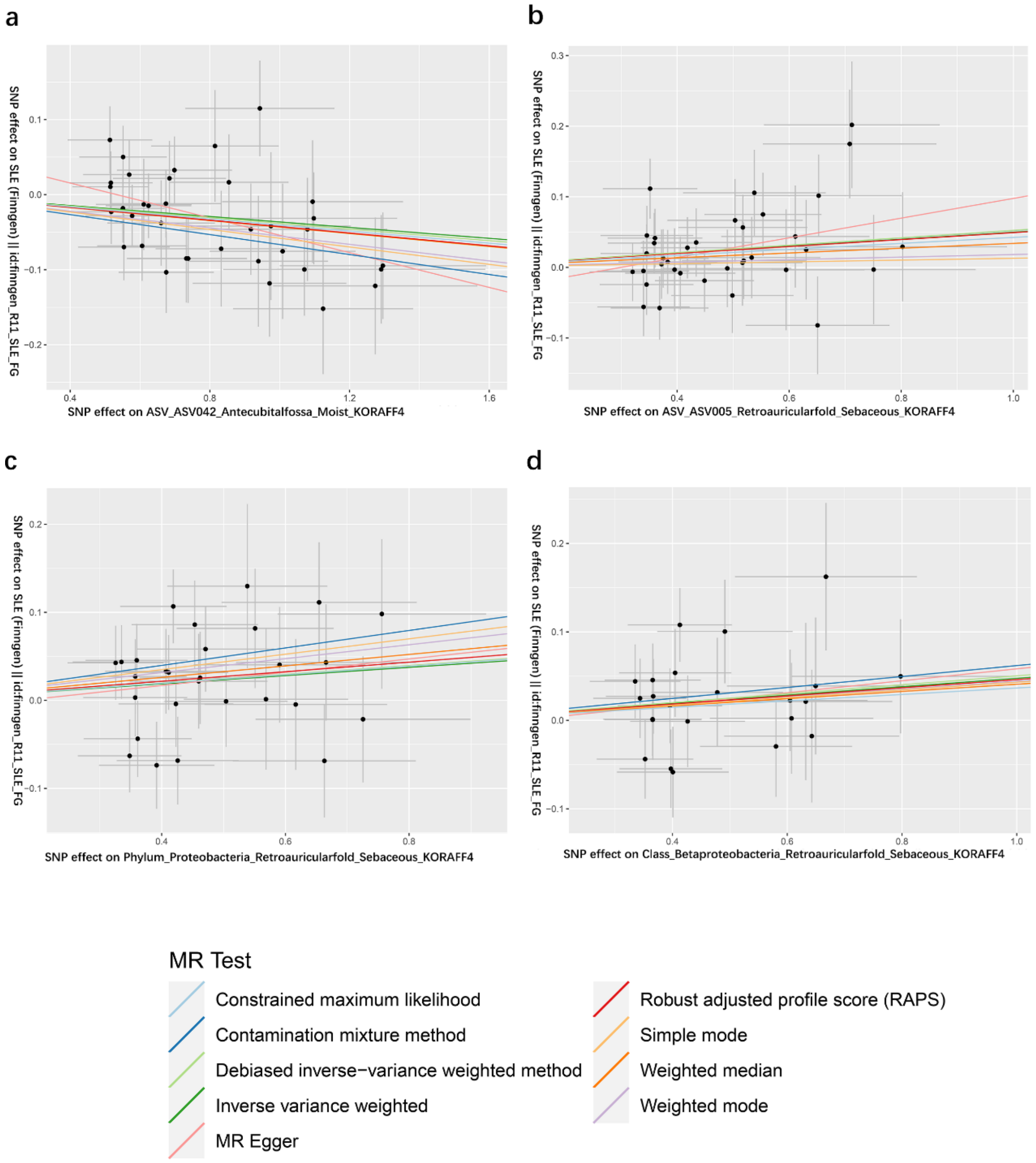

Supplementary Fig. S2 Scatter plot analysis of the association between skin microbiota (PopGen) and systemic lupus erythematosus. (a) ASV004[*Corynebacterium (unc.)*];(b) ASV005 [*Propionibacterium granulosum*];(c) ASV007[*Anaerococcus (unc.)*];(d) the genus *Kocuria*;(e) the class *Betaproteobacteria*;(f) ASV039 [*Acinetobacter (unc.)*].

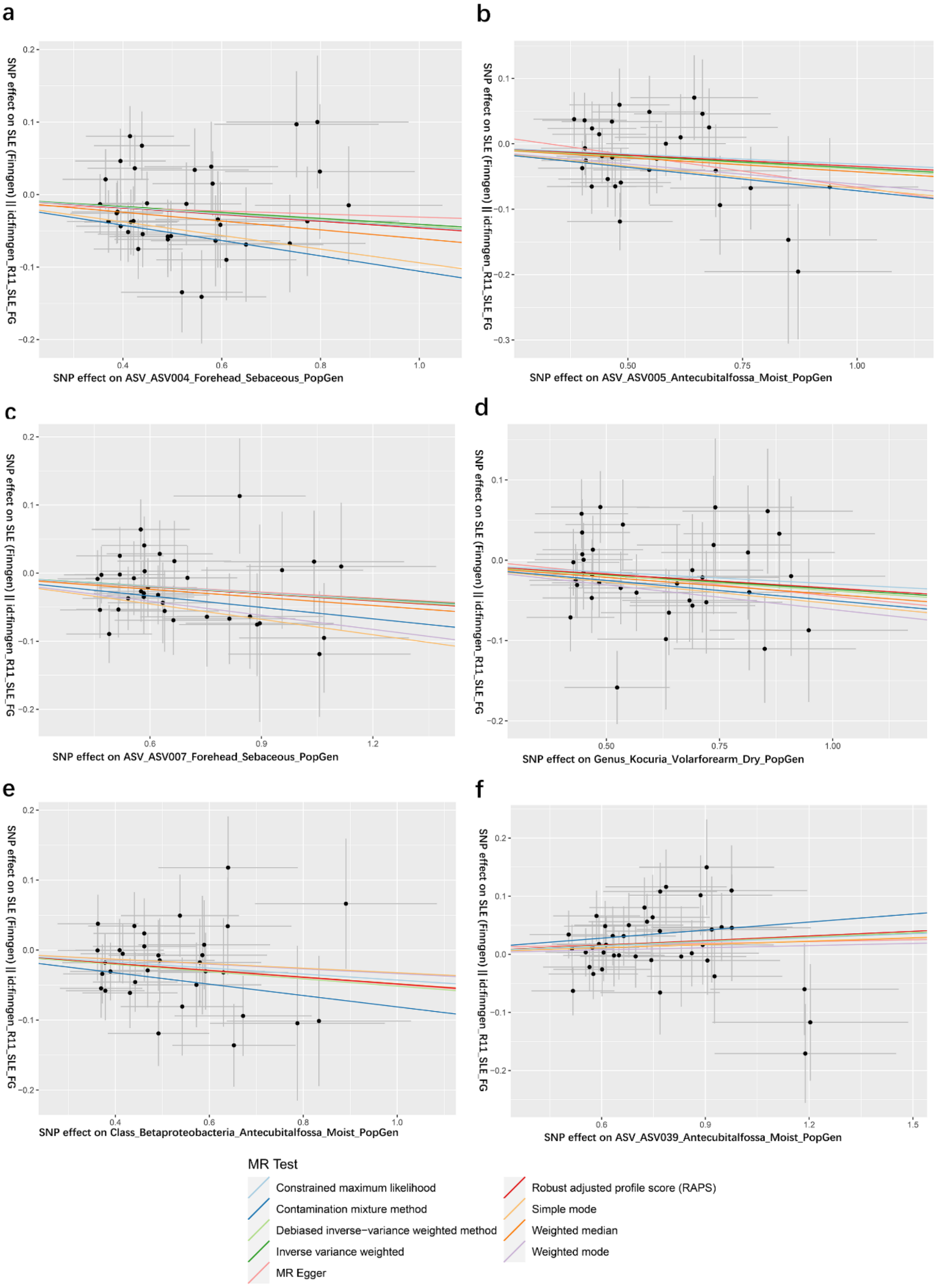

Supplementary Fig. S3 Forest plots of SNPs associated with skin microbiota (KORA FF4) and systemic lupus erythematosus. (a) ASV042 [Acinetobacter (unc.)];(b) ASV005 [*Propionibacterium granulosum*];(c) the phylum *Proteobacteria*;(d) the class *Betaproteobacteria*.

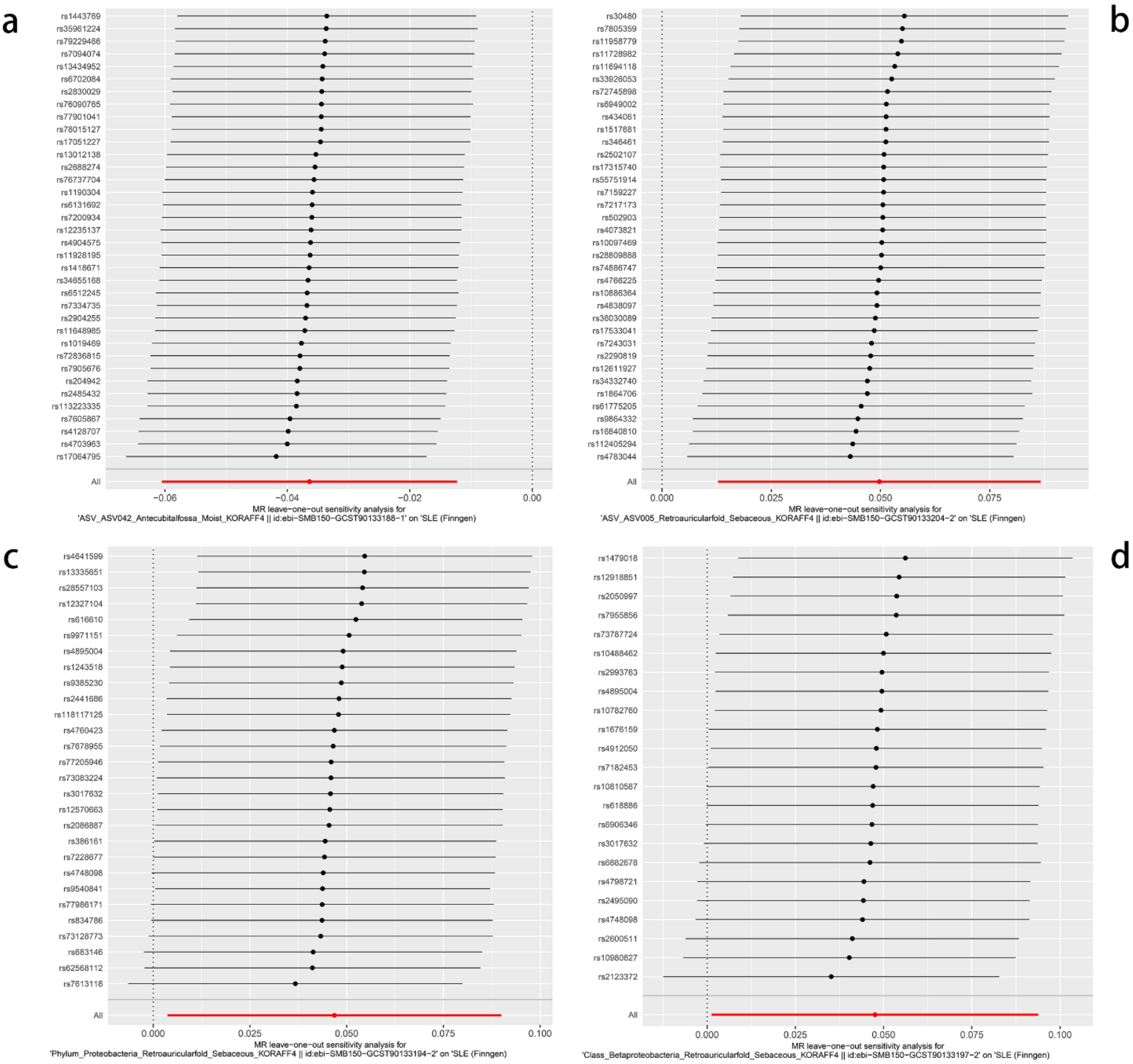

Supplementary Fig. S4 Forest plots of SNPs associated with skin microbiota (PopGen) and systemic lupus erythematosus. (a) ASV004[*Corynebacterium (unc.)*];(b) ASV005 [*Propionibacterium granulosum*];(c) ASV007[*Anaerococcus (unc.)*];(d) the genus *Kocuria*;(e) the class *Betaproteobacteria*;(f) ASV039 [*Acinetobacter (unc.)*].

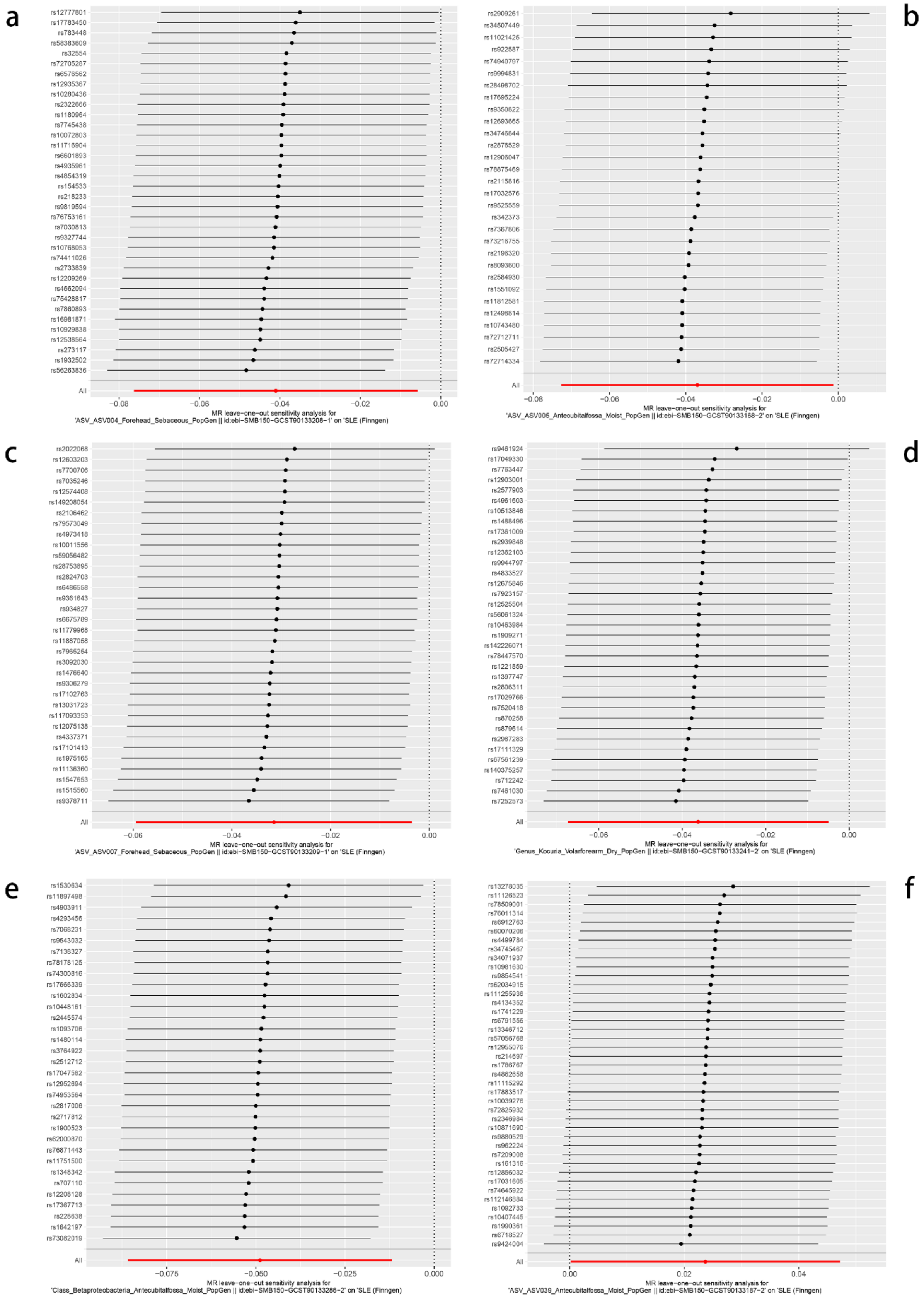

Supplement: Supplementary file 2 — (PDF 1.40 MB) [file 10067_2025_7556_MOESM2_ESM.pdf]
